# Clinical Research on the Safety Evaluation of Platelet-rich Plasma Treatment in Oral Diseases: A Study Protocol

**DOI:** 10.14789/jmj.JMJ23-0005-SP

**Published:** 2023-04-26

**Authors:** MORIKUNI TOBITA, YOSUKE MASUBUCHI, KEIKO WAKANA, HIKARI YONEDA, SHUNSUKE NAMAKI, MARIKO HIDE, TAKAAKI TAMAGAWA, MITSUYO SHINOHARA

**Affiliations:** 1 Department of Oral and Maxillofacial Surgery, Juntendo University Hospital, Tokyo, Japan; 1 Department of Oral and Maxillofacial Surgery, Juntendo University Hospital, Tokyo, Japan; 2 Research Office for Regulatory Science and Research Ethics, Medical Technology Innovation Center, Juntendo University, Tokyo, Japan; 2 Research Office for Regulatory Science and Research Ethics, Medical Technology Innovation Center, Juntendo University, Tokyo, Japan; 3 Clinical Trial and Research Center, Juntendo University Hospital, Tokyo, Japan; 3 Clinical Trial and Research Center, Juntendo University Hospital, Tokyo, Japan; 4 Department of Oral and Maxillofacial Surgery Ⅱ, Nihon University School of Dentistry, Tokyo, Japan; 4 Department of Oral and Maxillofacial Surgery Ⅱ, Nihon University School of Dentistry, Tokyo, Japan; 5 Department of Oral and Maxillofacial Surgery, Juntendo University Nerima Hospital, Tokyo, Japan; 5 Department of Oral and Maxillofacial Surgery, Juntendo University Nerima Hospital, Tokyo, Japan

**Keywords:** platelet-rich plasma, clinical research, safety, dental treatment

## Abstract

**Background:**

Platelet-rich plasma (PRP) is a biological product obtained from autologous blood that contains growth factors, promoting the healing and regeneration of human tissues. Several oral diseases require surgical intervention, producing residual wounds that undergo a healing process, accompanied by pain, swelling, superinfections, and bone remodeling. This protocol study aims to evaluate the safety of PRP use for the following dental procedures: post-extraction socket healing, periodontal tissue regeneration, maxillary sinus floor elevation, tooth transplantation, and intentional tooth replantation.

**Methods:**

Ten patients will be enrolled and subjected to the required treatment with the addition of PRP, after appropriate hematological and biochemical evaluations. The participants will then be subjected to an observation period of 4 weeks to monitor adverse events through clinical observation. Secondary outcomes will regard pain, and clinical evolution of the treated site. Among these, presence of infection, swelling, wound healing, stability of the transplanted tooth.

**Discussion:**

Safety of medical procedures represents the first requirement for their introduction in routine practice. A careful evaluation of clinical response during follow-up period and registration of adverse effects is fundamental for safety confirmation and subsequent use of PRP for the proposed dental procedures.

**Trial registration:**

Japan Registry of Clinical Trials (https://jrct.niph.go.jp/, registry number: jRCTc030190273, jRCTc030190274, jRCTc030190275, jRCTc030190276, jRCTc030190277; Date of registration: 31 March 2020).

## Background

### Introduction to the trial

Clinical research on the safety evaluation of platelet-rich plasma (PRP) use in oral procedures: wound healing after tooth extraction, maxillary sinus floor elevation surgery, tooth transplantation, intentional tooth replantation, and periodontal tissue regeneration.

### Background and rationale

Platelet-rich plasma (PRP) is derived from venous blood and has been proposed for several clinical applications^1, 2)^. The use of platelet concentrate is at the center of a recent academic debate, therefore new clinical studies are needed to prove its efficacy^3)^. In particular, its activity has been demonstrated for the treatment of chronic wounds and tissue repair^4)^. The most diffused technique for obtaining PRP is to use autologous blood (from the patients themselves) and centrifugation to separate red blood cells from plasma and white blood cells. The obtained highly concentrated platelets contain several growth factors, including platelet-derived growth factor, epithelial growth factor, and fibroblast growth factor, as well as some other molecules involved in regenerative processes^5, 6)^.

Its use has been proposed for the treatment of chronic ulcers^7)^, osteoarthritis, and degenerative diseases^8)^, demonstrating promising results in terms of promoting healing, pain reduction, and functional improvement.

The oral cavity comprises soft and hard tissues that may require surgical interventions for the treatment of tooth-related or periodontium-related diseases to replace missing teeth. These procedures produce wounds that are characterized by typical inflammatory reactions that lead to more or less rapid healing with different outcomes, including a certain rate of bone remodeling. The use of PRP for dental procedures has been proposed by several authors^9)^ to enhance soft tissue healing and bone regeneration. Furthermore, it has been debated if the use of PRP can improve the prevention and treatment of medication-related osteonecrosis of the jaws^10)^. The reported results vary depending on the type of procedure, method of PRP preparation, observation period, and evaluated outcomes^11-15)^. Furthermore, little evidence is available regarding the safety (infection risk, systemic complications, and enhanced inflammation) of PRP use in dentistry.

This study aims to present a protocol for safety evaluation of PRP use in the following dental procedures: 1) post-extraction sockets, 2) periodontal regenerative therapy, 3) maxillary sinus lift, 4) tooth transplantation, and 5) tooth replantation. The rationale and description of the procedures are briefly described as follows:

#### Wound healing of the post-extraction socket

Dental extractions are the most common surgical procedures in the oral cavity. Such a procedure can be more or less traumatic, and the post-extraction socket is often left uncovered when flap closure is not feasible. The healing process comprises a series of complex changes involving hard and soft tissues referred to as “socket healing.” In this process, the following three sequential phases can be identified: inflammatory, proliferative, and modeling/remodeling^16)^. Considering the biological activity of PRP, the hypothesis is that its application in post-extraction sockets may accelerate and improve the healing process.

#### Periodontal tissue regeneration

Periodontal disease is considered as the most common infection worldwide^17)^. Its pathogenic process leads to the progressive destruction of tooth-supporting tissues. The treatment of periodontitis involves the adoption of well-established protocols of oral hygiene and surgical and non-surgical interventions. Tissue destruction caused by periodontitis is often irreversible; nevertheless, some specific conditions, if properly treated, can be regenerated^18)^. Tissue engineering technologies are fundamental for this purpose, and continuous research exists on materials that can promote the regeneration of tissues surrounding the teeth^19-21)^.

#### Maxillary sinus floor elevation

Another widely diffused procedure nowadays is the dental implant rehabilitation of missing teeth. To perform this intervention, a sufficient amount of bone is required. In some cases, the close proximity of adjacent anatomical structures can limit the possibility of dental implant insertion. The maxillary sinus represents one of these, and sometimes, procedures of sinus floor elevation are needed before dental implant placement. Therefore, different techniques and materials have been proposed for the sinus lift, including various types of bone substitutes^22-26)^.

As part of these well-standardized and predictable procedures, some others have not been sufficiently experimented and are currently considered uninsured treatments. Among these, the tooth transplantation and intentional tooth replantation are becoming more and more investigated.

#### Tooth transplantation

Tooth transplantation (or tooth grafting) is a method that comprises transferring a tooth from another part to an area where a tooth has been lost due to dental caries or periodontal disease. In general, wisdom teeth are often used because they have less impact on occlusion^27)^. The 10-year survival rate of the transplanted tooth was reported to be 73.6%^28, 29)^.

#### Intentional tooth replantation

Dislocated teeth due to trauma or other reasons can be relocated to their original position through replantation. Tooth grafting is expected to last for several years after surgery, allowing for natural function.

The use of PRP in these and other oral procedures has been proposed by several authors, showing promising results^8)^. Nevertheless, there is still a concern regarding the appropriateness of providing regenerative medicine as a medical treatment, since the evaluation method and the method of provision have not been sufficiently verified. Additionally, the benefit to the patient must outweigh the risk when regenerative medicine is used as a treatment^30, 31)^.

## Methods/Design

### Study design

This single-arm open-label study is ongoing and is being conducted from March 2019 to March 2024 at the Department of Dental Surgery, Juntendo University Hospital, Juntendo, Japan. The study protocol was approved by the Tokyo Medical and Dental University Specially Certified Committee for Regenerative Medicine (committee number: NA8140003, approval number: RM2018-008, RM2018-09, RM2018-010, RM2018-011, RM2018-012) and has been registered in the Japan Registry of Clinical Trials (https://jrct.niph.go.jp/, registry number: jRCTc030190273, jRCTc030190274, jRCTc 030190275, jRCTc030190276, jRCTc030190277).

### Eligibility criteria

A list of common and treatment-specific inclusion criteria is established. In particular, the common inclusion criteria are as follows:

1. A good systemic condition without chronic or acute diseases.

2. Number of platelets above 1x10E5/µL;

3. Aged ≥20 years;

4. Signed informed consent.

Treatment-specific requirements for enrollment in one of the protocols of the study (1–5) are as follows:

1. Having a wisdom tooth requiring extraction.

2. Having a periodontal pocket of >5 mm at baseline examination with an intrabony defect of ≥5 mm depth and ≥2 mm width at the interproximal site, as observed using radiographs. The mobility of experimental tooth has to not exceed grade 2, with availability of keratinized gingiva, good oral hygiene and the tooth must not require surgical/restorative/root canal treatment within 36 weeks after PRP transplantation.

3. Having a missing tooth in the maxillary posterior region requiring dental implant rehabilitation and sinus floor augmentation.

4. Having a molar tooth that needs to be extracted and having a wisdom tooth that can be extracted.

5. Having a fracture of the dental root for which replantation is a viable treatment option and no abscess at the root is present.

The exclusion criteria are defined as follows:

1. Patients suspected of or having a history of complicated malignant tumors;

2. Presence of or a history of abnormal gingival proliferation;

3. Presence of anti-coagulant or anti-platelet medications or bleeding disorders;

4. Pregnancy, breastfeeding or intention of pregnancy;

5. Alcoholism or drug dependence

6. Presence of hepatitis C virus (HCV) antibody, hepatitis B surface (HBs) antigen, adult T-cell leukemia virus-associated antigen virus antibody, or human immunodeficiency virus (HIV) antibody

### Planned sample size

Recruitment of 10 patients (two per treatment) is planned.

### Study procedures

All patients will be provided with a consent document approved by an authorized committee for regenerative medicine. Every oral and written explanation will be provided, and written voluntary consent will be obtained from the participants. Patients satisfying the inclusion criteria will be subjected to a hematological examination:

1. White blood cell count, white blood cell fractions (neutrophils, eosinophils, basophils, and lymphocytes), red blood cell count, hematocrit, hemoglobin, and platelet count.

2. Blood biochemical tests: Aspartate aminotransferase (glutamic-oxaloacetic transaminase), alanine aminotransaminase (glutamic-pyruvic transaminase), total protein, and creatinine.

3. Viral tests: HCV antibody, HBs antigen, and HIV antibody.

Observation and investigation items are summarized in Table 1.

**Table 1:** Observation, investigation, and evaluation items

Item	Description
Patient background	Date of obtaining consent, sex, date of birth, name of the causative disease, complications and their severity, preexisting medical conditions, presence of infectious diseases, history of dental surgery, and concomitant treatment.
Vital signs	Blood pressure (maximum, minimum); heart rate; and body temperature
Clinical examination	Subjective symptoms
Hematological examination	White blood cell count; white blood cell fraction (neutrophils, eosinophils, basophils, lymphocytes); red blood cell count; Hct; Hb; and platelet count
Blood biochemical tests	AST (GOT), ALT (GTP), T-P, and CRE
Adverse events	The presence/absence, timing, resolution, extent, treatment, severity assessment, and relevance to the cell product in question of any adverse events observed during the study.
Concomitant medications	Type, dose, and timing of concomitant medications used during the study.
Pain	Interviews about the onset time of pain, type and degree of pain, duration of analgesia medications, and degree of pain control, rapidity, and persis-tence.
Sterility test	Sterility test using a portion of gelatinized PRP for transplantation.
Intraoral photography	Intraoral photography before and after treatment using a digital camera.
Dental radiography	Assessment of infection signs.
Platelet concentration ratio	Platelet count (x10^5^/μL), red blood cell count (x10^4^/μL), and white blood cell count (x10^3^/μL) in whole blood and prepared PRP before centrifugation using automated hematology analyzer.

Hct, hematocrit; Hb, hemoglobin; AST (GOT), aspartate aminotransferase (glutamic-oxaloacetic transaminase); ALT (GPT), alanine aminotransaminase (glutamic-pyruvic transaminase); T-P, total protein; CRE, creatinine

After enrollment, on the day of treatment, 26 mL of blood will be collected from the participants' mid-elbow vein using a syringe containing anticoagulant (anticoagulant citrate dextrose solution) as a blood sample for PRP preparation^32)^. Approximately 1 mL of the collected peripheral blood will be used to produce PRP at the Juntendo University cell culture and processing facility. A visual test and inspection of the foreign matter in the fabricated PRP will be performed. Furthermore, the platelet concentration ratio will be counted in whole blood and PRP will be prepared before centrifugation using an automated hematology analyzer. A sterility test will be performed on gelatinized PRP to verify its sterility.

Approximately one-tenth of the volume of 2% CaCl_2_ and, if necessary, autologous thrombin will be added to 1 mL of PRP to gel it immediately before use.

Simultaneously with PRP preparation, the appropriate dental procedure will be performed, and PRP gel will be used as follows:

1. After tooth extraction, the post-extraction socket will be filled with PRP and, if possible, the flaps will be approximated using a resorbable surgical suture.

2. In the periodontal flap surgery, after open-flap debridement, PRP will be applied on the tooth surface involved in bone defects, and the flap will be approximated using a resorbable suture.

3. For sinus lift, after the elevation of the flap and access to the sinus will be obtained using a piezoelectric instrument. After that, the sinus membrane will be elevated and PRP mixed with the bone graft will be applied. The mucoperiosteal flap will be sutured then.

4. In tooth transplantation and replantation, gelatinized PRP will be administered in the tooth graft site, and the grafted tooth will be implanted. The transplanted or replanted tooth will be temporarily fixed to the tooth adjacent to the dental resin.

After the treatment, the participants will be evaluated for safety for 4 weeks and will be followed up for 11 months (48 weeks).

### Primary endpoint: Safety evaluation

Adverse effects will be evaluated in terms of presence/absence, timing, resolution, extent, treatment, and severity. Adverse events will be evaluated during the 4-week period of transplantation by clinical examination of subjective and objective symptoms and encoded according to the Common Terminology Criteria for Adverse Events^33)^; an adverse event of grade 3 or higher is suspected to be related to the provision of regenerative medicine, and a consideration of whether or not to continue enrollment in the study will be undertaken. Furthermore, the enrollment of the second case will be suspended until the safety evaluation of the first case is completed.

#### Secondary endpoints

1. Interviews for postoperative pain. Changes in postoperative pain will be evaluated during the observational period (4 weeks) using a 10- point numerical rating scale. Interviews will be conducted to determine the pain onset time, type, and degree; duration of analgesic medication; and degree of pain control, rapidity, and persistence.

2. Clinical and radiologic healing will be evaluated using intraoral photography, endoral radiography, and cone-beam computed tomography. The following items will be evaluated for each treatment:

•Presence of infection;

•Swelling;

•Wound healing;

•Stability of the transplanted tooth.

3. Correlation between the platelet enrichment rate of PRP and outcome.

## Discussion

The use of PRP can represent a breakthrough in regenerative dentistry because of its relatively simple and inexpensive extraction and application procedures. Its potential fields of application include several branches of dentistry, such as extractions, dental implant rehabilitation, periodontal treatment, and transplantation. The results of this study will provide additional data regarding the safety of PRP use for dental procedures and provide accurate data to both clinicians and patients.

## Funding

This research received no external funding.

## Author contributions

Conceptualization, MT; methodology, YM; software, YM; validation, KW; formal analysis, MT; investigation, MT, SN, MH, TT, and MS; resources, MT; data curation, KW and HY; writing—original draft preparation, MT; writing—review and editing, MT; visualization, YM; supervision, MT; project administration, YM and KW. All authors read and approved the final manuscript.

## Conflicts of interest statement

The authors declare that there are no conflicts of interest.
